# The lower limit of reactivity as a potential individualised cerebral perfusion pressure target in traumatic brain injury: a CENTER-TBI high-resolution sub-study analysis

**DOI:** 10.1186/s13054-023-04485-8

**Published:** 2023-05-20

**Authors:** Erta Beqiri, Frederick A. Zeiler, Ari Ercole, Michal M. Placek, Jeanette Tas, Joseph Donnelly, Marcel J. H. Aries, Peter J. Hutchinson, David Menon, Nino Stocchetti, Marek Czosnyka, Peter Smielewski, CENTER-TBI HR ICU participants and investigators, Audny Anke, Ronny Beer, Bo-Michael Bellander, Erta Beqiri, Andras Buki, Manuel Cabeleira, Marco Carbonara, Arturo Chieregato, Giuseppe Citerio, Hans Clusmann, Endre Czeiter, Marek Czosnyka, Bart Depreitere, Ari Ercole, Shirin Frisvold, Raimund Helbok, Stefan Jankowski, Daniel Kondziella, Lars-Owe Koskinen, Ana Kowark, David K. Menon, Geert Meyfroidt, Kirsten Moeller, David Nelson, Anna Piippo-Karjalainen, Andreea Radoi, Arminas Ragauskas, Rahul Raj, Jonathan Rhodes, Saulius Rocka, Rolf Rossaint, Juan Sahuquillo, Oliver Sakowitz, Peter Smielewski, Nino Stocchetti, Nina Sundström, Riikka Takala, Tomas Tamosuitis, Olli Tenovuo, Andreas Unterberg, Peter Vajkoczy, Alessia Vargiolu, Rimantas Vilcinis, Stefan Wolf, Alexander Younsi, Frederick A. Zeiler

**Affiliations:** 1 Brain Physics Laboratory, Division of Neurosurgery, Department of Clinical Neurosciences, University of Cambridge, Cambridge, UK; 2 Department of Surgery, Rady Faculty of Health Sciences, University of Manitoba, Winnipeg, MB Canada; 3 Section of Neurosurgery, Department of Surgery, Rady Faculty of Health Sciences, University of Manitoba, Winnipeg, MB Canada; 4 Department of Human Anatomy and Cell Science, Rady Faculty of Health Sciences, University of Manitoba, Winnipeg, Canada; 5 Biomedical Engineering, Price Faculty of Engineering, University of Manitoba, Winnipeg, Canada; 6 Division of Anaesthesia, Department of Medicine, University of Cambridge, Cambridge, UK; 7 Department of Clinical Neuroscience, Karolinska Intitutet, Stockholm, Sweden; 8 School for Mental Health and Neuroscience (MHeNS), University Maastricht, Maastricht, The Netherlands; 9 Department of Intensive Care Medicine, Maastricht University Medical Center+, Maastricht, The Netherlands; 10 Department of Medicine, University of Auckland, Auckland, New Zealand; 11 Division of Neurosurgery, Department of Clinical Neurosciences, Addenbrooke’s Hospital and University of Cambridge, Cambridge, CB2 0QQ UK; 12 Neuroscience Intensive Care Unit, Department of Anaesthesia and Critical Care, Fondazione IRCCS Ca’ Granda-Ospedale Maggiore Policlinico, Milan, Italy; 13 Department of Pathophysiology and Transplants, University of Milan, Milan, Italy; 14 Department of Physical Medicine and Rehabilitation, University Hospital Northern Norway, Tromsø, Norway; 15 Department of Neurology, Neurological Intensive Care Unit, Medical University of Innsbruck, Innsbruck, Austria; 16 Department of Neurosurgery & Anesthesia & Intensive Care Medicine, Karolinska University Hospital, Stockholm, Sweden; 17 NeuroIntensive Care, Niguarda Hospital, Milan, Italy; 18 Department of Neurosurgery, Medical School, University of Pécs, Pécs, Hungary; 19 Neurotrauma Research Group, János Szentágothai Research Centre, University of Pécs, Pécs, Hungary; 20 Brain Physics Lab, Division of Neurosurgery, Department of Clinical Neurosciences, University of Cambridge, Addenbrooke’s Hospital, Cambridge, UK; 21 Neuro ICU, Fondazione IRCCS Cà Granda Ospedale Maggiore Policlinico, Milan, Italy; 22 NeuroIntensive Care Unit, Department of Anesthesia & Intensive Care, ASST di Monza, Monza, Italy; 23 School of Medicine and Surgery, Università Milano Bicocca, Milan, Italy; 24 Department of Neurosurgery, Medical Faculty, RWTH Aachen University, Aachen, Germany; 25 Department of Neurosurgery, University of Pecs, Pécs, Hungary; 26 MTA-PTE Clinical Neuroscience MR Research Group and Janos Szentagothai Research Centre, University of Pecs, Hungarian Brain Research Program (Grant No. KTIA 13 NAP-A-II/8), Pecs, Hungary; 27 Department of Neurosurgery, University Hospitals Leuven, Louvain, Belgium; 28 Department of Anesthesiology and Intensive Care, University Hospital Northern Norway, Tromso, Norway; 29 Neurointensive Care, Sheffield Teaching Hospitals NHS Foundation Trust, Sheffield, UK; 30 Departments of Neurology, Clinical Neurophysiology and Neuroanesthesiology, Region Hovedstaden Rigshospitalet, Copenhagen, Denmark; 31 Department of Clinical Neuroscience, Neurosurgery, Umeå University, Umeå, Sweden; 32 Department of Anaesthesiology, University Hospital of Aachen, Aachen, Germany; 33 Intensive Care Medicine, University Hospitals Leuven, Louvain, Belgium; 34 Department Neuroanesthesiology, Region Hovedstaden Rigshospitalet, Copenhagen, Denmark; 35 Helsinki University Central Hospital, Helsinki, Finland; 36 Department of Neurosurgery, Vall d’Hebron University Hospital, Barcelona, Spain; 37 Department of Neurosurgery, Kaunas University of Technology and Vilnius University, Vilnius, Lithuania; 38 Department of Anaesthesia, Critical Care & Pain Medicine NHS Lothian & University of Edinburg, Edinburgh, UK; 39 Klinik Für Neurochirurgie, Klinikum Ludwigsburg, Ludwigsburg, Germany; 40 Department of Neurosurgery, University Hospital Heidelberg, Heidelberg, Germany; 41 Department of Radiation Sciences, Biomedical Engineering, Umea University, Umea, Sweden; 42 Perioperative Services, Intensive Care Medicine, and Pain Management, Turku University Central Hospital and University of Turku, Turku, Finland; 43 Neuro-Intensive Care Unit, Kaunas University of Health Sciences, Kaunas, Lithuania; 44 Rehabilitation and Brain Trauma, Turku University Central Hospital and University of Turku, Turku, Finland; 45 Neurologie, Neurochirurgie Und Psychiatrie, Charité – Universitätsmedizin Berlin, Berlin, Germany; 46 Department of Neurosurgery, Kaunas University of Health Sciences, Kaunas, Lithuania; 47 Department of Neurosurgery, Charité – Universitätsmedizin Berlin, Corporate member of Freie Universität Berlin, Humboldt-Universität Zu Berlin, and Berlin Institute of Health, Berlin, Germany

**Keywords:** Lower limit of reactivity, Cerebral autoregulation, Traumatic brain injury, Individualised cerebral perfusion pressure

## Abstract

**Background:**

A previous retrospective single-centre study suggested that the percentage of time spent with cerebral perfusion pressure (CPP) below the individual lower limit of reactivity (LLR) is associated with mortality in traumatic brain injury (TBI) patients. We aim to validate this in a large multicentre cohort.

**Methods:**

Recordings from 171 TBI patients from the high-resolution cohort of the CENTER-TBI study were processed with ICM+ software. We derived LLR as a time trend of CPP at a level for which the pressure reactivity index (PRx) indicates impaired cerebrovascular reactivity with low CPP. The relationship with mortality was assessed with Mann-U test (first 7-day period), Kruskal–Wallis (daily analysis for 7 days), univariate and multivariate logistic regression models. AUCs (CI 95%) were calculated and compared using DeLong’s test.

**Results:**

Average LLR over the first 7 days was above 60 mmHg in 48% of patients. %time with CPP < LLR could predict mortality (AUC 0.73, *p* =  < 0.001). This association becomes significant starting from the third day post injury. The relationship was maintained when correcting for IMPACT covariates or for high ICP.

**Conclusions:**

Using a multicentre cohort, we confirmed that CPP below LLR was associated with mortality during the first seven days post injury.

**Supplementary Information:**

The online version contains supplementary material available at 10.1186/s13054-023-04485-8.

## Introduction

The critical care management of traumatic brain injury (TBI) patients aims to reduce the occurrence and the burden of secondary insults, such as those caused by intracranial hypertension [1, 1]. Ensuring adequate cerebral perfusion pressure (CPP) is pivotal in supplying blood flow to the injured brain[3]. How to best individualise CPP targets in TBI patients admitted in intensive care unit (ICU) remains an open question.

Continuous monitoring of cerebral autoregulation (CA) could provide a way to tailor CPP targets, as proposed and pioneered by our research group [4–6]. The autoregulatory mechanism refers to the response of the cerebral circulation to changes in CPP. There is a range of CPP values where autoregulation actively minimises fluctuations in cerebral blood flow, when CPP is altered. Outside of this range, and on both sides, the relationship between pressure and flow becomes more passive. This leads to increased risk of both ischaemia (when CPP drops below the lower limit of autoregulation) or hyperaemia (when CPP goes beyond the upper limit of autoregulation) [3, 7]. A large body of studies has described the impairment of CA after TBI and the association between non-functioning CA and poor clinical outcome [8–10].

The pressure reactivity index PRx has been developed as a proxy for global CA in TBI patients with ICP monitoring [8, 11, 12]. PRx can be calculated in a semi-continuous manner in real time, at the bedside [13]. The relationship between PRx and values of CPP over past hours additionally provides information on the range of CPP over which CA is currently effective and whether the current CPP values are within the autoregulatory range [4, 14, 15][16]. The most investigated PRx-derived CPP target is the optimal CPP, named CPPopt [4, 14, 17]. By targeting CPP at the individualised CPPopt, in a dynamic manner, CA is best preserved [4] and it is plausible that this will offer protection against secondary injuries from sudden variations blood flow. This approach is undergoing clinical investigations [18, 19]. The results of the phase II trial (COGiTATE, registered as NCT02982122 in ClinicalTrials.gov) proved safety and feasibility of such application [20].

From the bed side point of view, the continuous assessment of the width and stability of the autoregulatory range might also be useful in addition to CPPopt [15, 21]. The range of autoregulation may vary over time due to disease progression. It might be very narrow, making CPPopt a desired target, or very large, in which case keeping CPP above the lower limit of autoregulation (LLA) could possibly be sufficient to prevent secondary injury. However, LLA is far less studied in the intensive care environment.

A statistically significant association with outcome of the deviation of CPP below LLA in TBI patients has been recently described by Donnelly et al. in a retrospective single-centre study [16]. The authors showed that the percentage of time (%time) spent with cerebral perfusion pressure (CPP) below the PRx-derived lower limit of autoregulation, named lower limit of reactivity (LLR), was independently positively associated with mortality in TBI patients. This suggested that targeting CPP above LLR, without the necessity of aiming for the optimal value (CPPopt), might be an option for the individualised clinical care in TBI patients.

LLR represents a global ensemble and requires more detailed methodological and clinical evaluation. Automated measurement of LLR (or the full autoregulatory range) is not straightforward: it extends but has received less attention than CPPopt methodologies. Robust continuous estimation requires further analytical development before clinical validation can be attempted.

﻿Here we validate an automated multiwindow-based algorithm for calculating LLR time trend in a multicentre cohort. We adapted the algorithm from the methodology used for prospective application of the CPPopt concept [22]. Our first objective was to assess the feasibility of the method. We hypothesised that the availability of the LLR time trend would be similar to the availability shown by CPPopt evaluated with the same methodology (CPPopt yield was estimated at 80.7% in this cohort [23]). Our second objective was to confirm in a multicentre cohort the positive association between mortality and the deviation of CPP below LLR. Finally, we sought to explore the outcome correlation in the light of the relationship between CPP below LLR and high ICP. It was recently suggested that the clinical burden of increased ICP is worse when PRx indicates an impaired autoregulation [24]. Given that LLR is derived from PRx, we expect this relationship to be maintained when looking at CPP below LLR.

## Material and methods

### Material

We considered 277 patients enrolled in the high-resolution cohort of the Collaborative European Neuro Trauma Effectiveness Research in TBI (CENTER-TBI) high-resolution ICU sub-study [25] over 21 recruiting centres from 2014 to 2017. All patients were admitted to ICU for their TBI during the course of the study. High-resolution digital signals were recorded from their ICU monitors during the course of their ICU stay.

The CENTER-TBI study (EC grant 602150) has been conducted in accordance with all relevant laws of the EU if directly applicable or of direct effect and all relevant laws of the country where the Recruiting sites were located, including but not limited to, the relevant privacy and data protection laws and regulations (the “Privacy Law”), the relevant laws and regulations on the use of human materials, and all relevant guidance relating to clinical studies from time to time in force including, but not limited to, the ICH Harmonised Tripartite Guideline for Good Clinical Practice (CPMP/ICH/135/95) (“ICH GCP”) and the World Medical Association Declaration of Helsinki entitled “Ethical Principles for Medical Research Involving Human Subjects”. Informed consent by the patients and/or the legal representative/next of kin was obtained, accordingly to the local legislations, for all patients recruited in the Core Dataset of CENTER-TBI and documented in the e-CRF.

Ethical approval was obtained for each recruiting site. The list of sites, Ethical Committees, approval numbers and approval dates can be found on the website: https://www.center-tbi.eu/project/ethical-approval

Data for the CENTER-TBI study has been collected through the Quesgen e-CRF (Quesgen Systems Inc, USA), hosted on the INCF platform and extracted via the INCF Neurobot tool (INCF, Sweden). For patient monitoring and data collection in the high-resolution repository, the ICM+ platform (University of Cambridge, UK) and/or Moberg Neuromonitoring system (Moberg Research Inc., USA) were used.

Detailed data collection and pre-processing methods (artefact cleaning and down-sampling to 10 s averaged time series) applied to high-resolution data of the cohort considered for our study have been described in preceding works [26, 27]. Arterial blood pressure (ABP) and intracranial pressure (ICP) 10-s averaged series were retrieved for this analysis.

The following demographic and low-resolution data were accessed using Opal software [28] on the 15th March 2021 and Neurobot version 3.0: age, sex, Glasgow Coma Scale (GCS), Marshall CT score, documented hypoxia or hypotension, length of ICU stay, decompressive craniectomy (DC), and Glasgow Outcome Scale Extended (GOSE) assessed at 6 months. We considered the first 7 days from the day of injury. We performed both a daily analysis and for the whole 7-day period.

Patients were considered eligible for our study if high-resolution data were available starting from the first 48 h from the day of injury (*n* = 253). We excluded patients with ICP recorded via the External Ventricular Drain (EVD, *n* = 35) and the ones that underwent DC (*n* = 29), as the ICP data quality and the validity of the PRx index in those cases is not fully established. Eighteen patients did not have GOSE outcome assessment at 6 months and their survival status is not known. Hence, the total number of patients included in the analysis was 171.

### Measurements

ICM + software [13, 29] was used for data processing. Lower limit of reactivity (LLR) minute-by-minute time trends were derived from ABP and ICP 10-s averages using the multiwindow-based algorithm adapted for prospective use as described in Beqiri et al. [22], where for our current research question the output variable was CPP at a certain threshold of PRx. PRx ranges from − 1 to + 1. The higher the PRx, the worse the cerebrovascular reactivity. Different values of PRx above 0 have been suggested as thresholds for identifying the lower breakpoint of the autoregulatory curve [30, 31]. In our study, when LLR was assessed using the PRx threshold of 0.2 or 0.4 the statistical results were similar. Therefore, we only report results for LLR at PRx = 0.2.

Minute-by-minute time trend values of CPP, LLR and ICP were averaged for further analysis. For each of these variables, one average value over 7 days was calculated for each patient for the whole 7-day period analysis. Conversely, one average value for each day and for each patient was considered for the daily analysis.

**Fig. 1:**
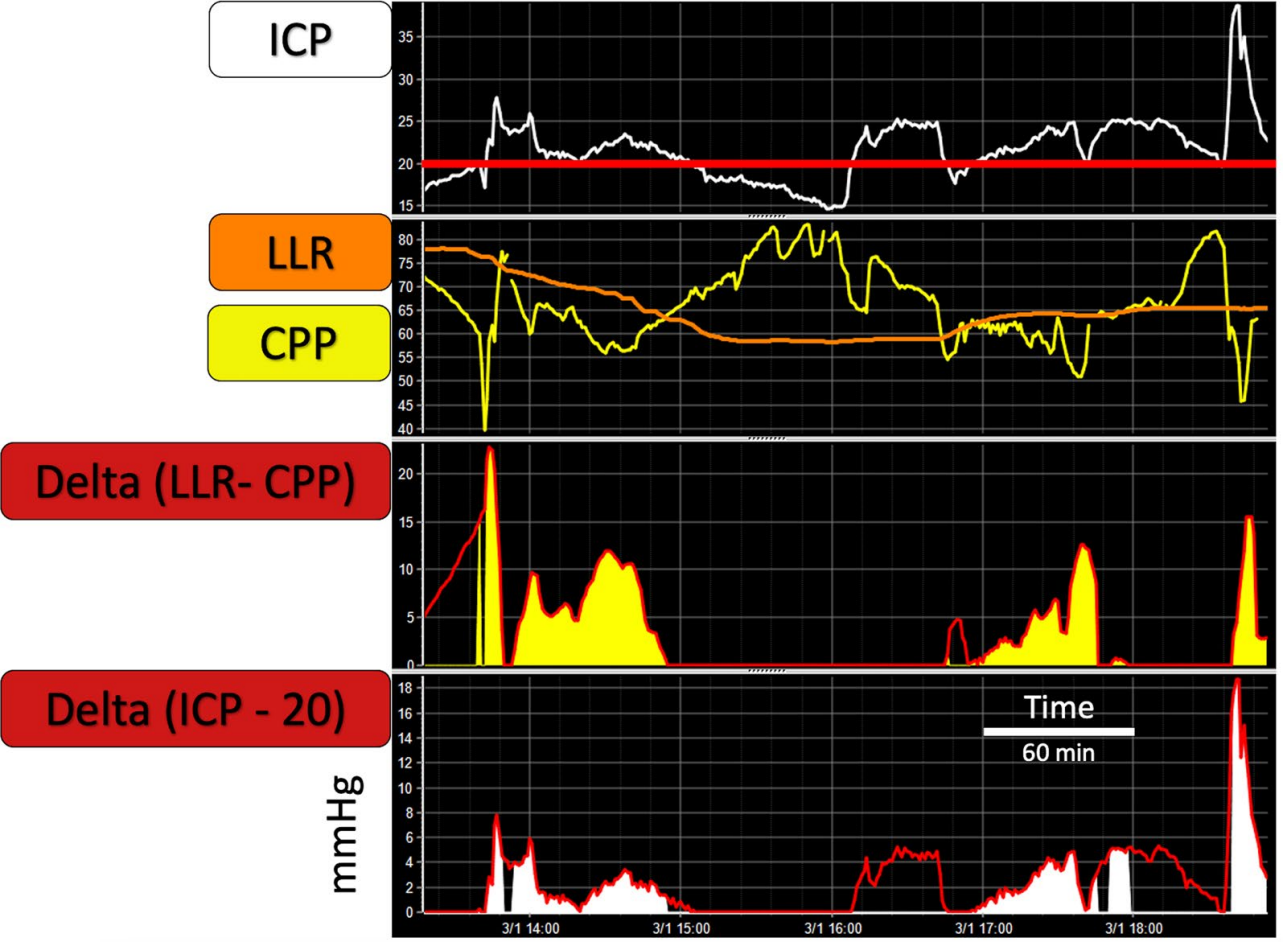
Min-by-min time trends of metrics used for studying the relationship between CPP < LLR and high ICP. The first top chart shows an example of ICP (in white) pressure–time chart. The horizontal red line identifies ICP at 20 mmHg. The second top chart shows LLR (orange) and CPP (yellow) time trends. Note that this example captures a highly dynamic time trend of LLR, which can perhaps be viewed as controversial. However, given an equally dynamic nature of TBI pathology within its acute stage, and a growing appreciation of fragility of the CA mechanism, plausibility of such an event cannot be denied. Unfortunately, owing to the lack of other relevant measurements in this case (eg CO_2_), explanation for this pattern could not be fully explored. The third chart shows in red the trend for delta CPP below LLR. Delta is calculated as ‘LLR – CPP’ and shown as absolute value. The area in yellow represents Dose of CPP below LLR only when ICP is above the threshold of 20 mmHg. The bottom chart shows in red the trend for delta ICP above 20 mmHg. Delta is calculated as ‘ICP – 20’ and showed as absolute value. The area in white represents dose of ICP above 20 mmHg only when CPP is below LLR. ICP: intracranial pressure; LLR: lower limit of reactivity; CPP: cerebral perfusion pressure

For the feasibility objective, we calculated availability (number of patients with LLR available at any point within the period considered) and yield (% of CPP recorded time with LLR available). The relationship between CPP and LLR was assessed using a metric of delta (i.e. deviation of) CPP below LLR (mmHg), dose of CPP below LLR (mmHg*h), and the cumulative time period with CPP below LLR, relative to the total available CPP data period (%). The relationship with ICP was explored using dose of CPP below LLR when ICP was above different thresholds (20–22–25 mmHg), and with dose of ICP above different thresholds when CPP is below LLR, as explained in Fig. 1. Statistical results for the different ICP thresholds were similar; therefore, only results for the threshold of 20 mmHg are reported in the multivariate analysis.

### Statistical analysis

Normality of continuous variables was assessed with histograms, quantile–quantile plots and Shapiro-Wilks test. Outcome groups were identified using the GOSE score [32]. Mortality was defined by GOSE = 1. The relationship with dichotomised outcome (dead vs alive) was assessed with Mann-U test (whole 7-day period, nonparametric test for independent samples), Kruskal–Wallis (daily analysis, nonparametric test for multiple-group comparison), univariate and multivariate logistic regression models. International Mission for Prognosis and Analysis of Clinical Trials (IMPACT) core [33] variables were considered for baseline characteristics adjustment in multivariate models. Backward stepwise elimination was performed on multivariate models when appropriate. AUC (CI 95%) were calculated and compared with the DeLong Test.

For completeness, the relationship with outcome dichotomised for distinguishing favourable and unfavourable outcome groups was also explored. Unfavourable outcome was defined with GOSE < 4.

## Results

Table 1 shows descriptive statistics of demographic, injury severity, admission and outcome variables of the 171 patients included in the analysis.

**Table 1:** Descriptive statistics of demographic, injury severity, admission and outcome variables

Variable	N or Median	% or IQR
Age		
Age (yrs)	53	(36–65)
Sex		
F	36	21
M	135	79
GCS		
3–8	108	63
9–13	34	20
14–15	21	12
GCS Motor score		
1	52	30
2	10	6
3	16	9
4	22	13
5	44	26
6	24	14
Pupils		
Both reactive	118	69
One reactive	14	8.2
Both unreactive	30	17.5
Hypoxia		
No	127	74
Definite	15	9
Suspect	4	2
Unknown	25	15
Hypotension		
No	118	69
Definite	19	11
Suspect	9	5
Unknown	25	15
Marshall CT score		
Diffuse injury I	5	2.9
Diffuse injury II	63	36.8
Diffuse injury III (swelling)	14	8.2
Diffuse injury IV (shift)	3	1.8
Evacuated mass lesion V	2	1.2
Non-evacuated mass lesion VI	59	34.5
Duration of neuromonitoring		
Duration of the recordings (days)	5	(3.3–5.6)
Length of Stay		
ICU Length of Stay (days)	13	(8–20)
GOSE 6 months		
1	38	22.2
3	47	27.5
4	9	5.3
5	37	21.6
6	18	10.5
7	12	7
8	10	5.8

*GCS* Glasgow Coma Scale, *CT* computerised tomography, *ICU* intensive care unit, *GOSE* Glasgow Outcome Scale Extended, *IQR* interquartile range

Note 1: patients with GCS 14–15 (mild TBI) received ICP monitoring after deterioration leading to ICU admission for care and monitoring

Note 2: GOSE 2 and 3 were combined in this dataset

### Feasibility

**Fig. 2:**
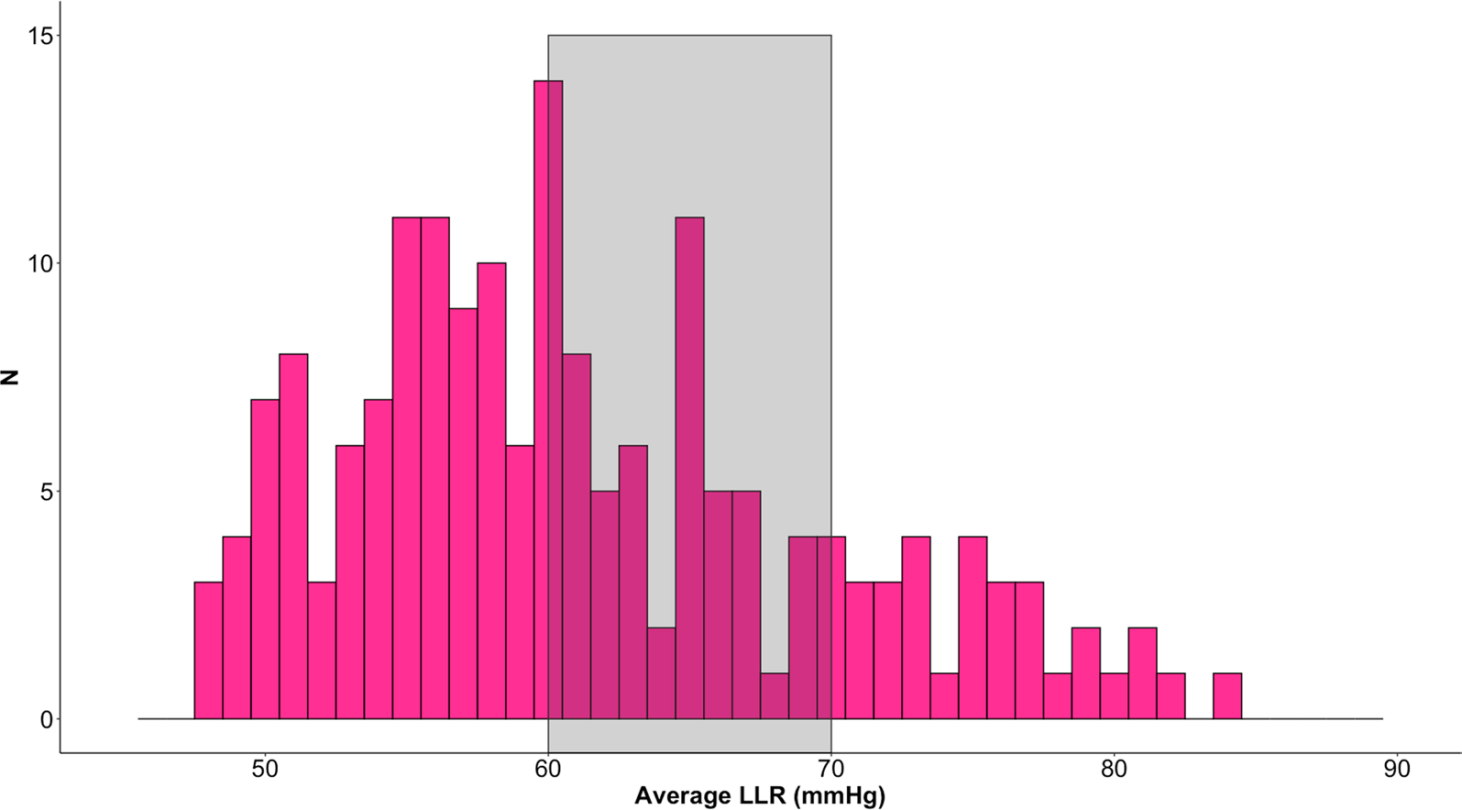
Distribution of average LLR for the first 7 days post injury. For each patient, the average value of LLR over the 7-day period from the day of injury was calculated. The histogram shows the distribution of the values so obtained. The grey rectangle highlights the fixed range recommended for cerebral perfusion pressure management by international guidelines. LLR: Lower limit of reactivity. N: Number of patients

The lower limit of reactivity (LLR) was available in 169 patients (98.8%); the two patients with no discernible LLR had very high PRx (average values of 0.95 and 0.92) and un-survivable ICP (average values of 62 and 105 mmHg) from the very beginning of the recording. Median (IQR) LLR yield was 79.8% (68.8–87.4). Figure 2 shows the population distribution of LLR values over the first 7 days from injury. For 87 patients (51.5%), LLR average value for the 7-day period was below 60 mmHg. Thirty-one patients (18.3%) had average LLR values above 70 mmHg.

### Outcome analysis for the whole 7-day period

#### Univariate analysis

**Fig. 3:**
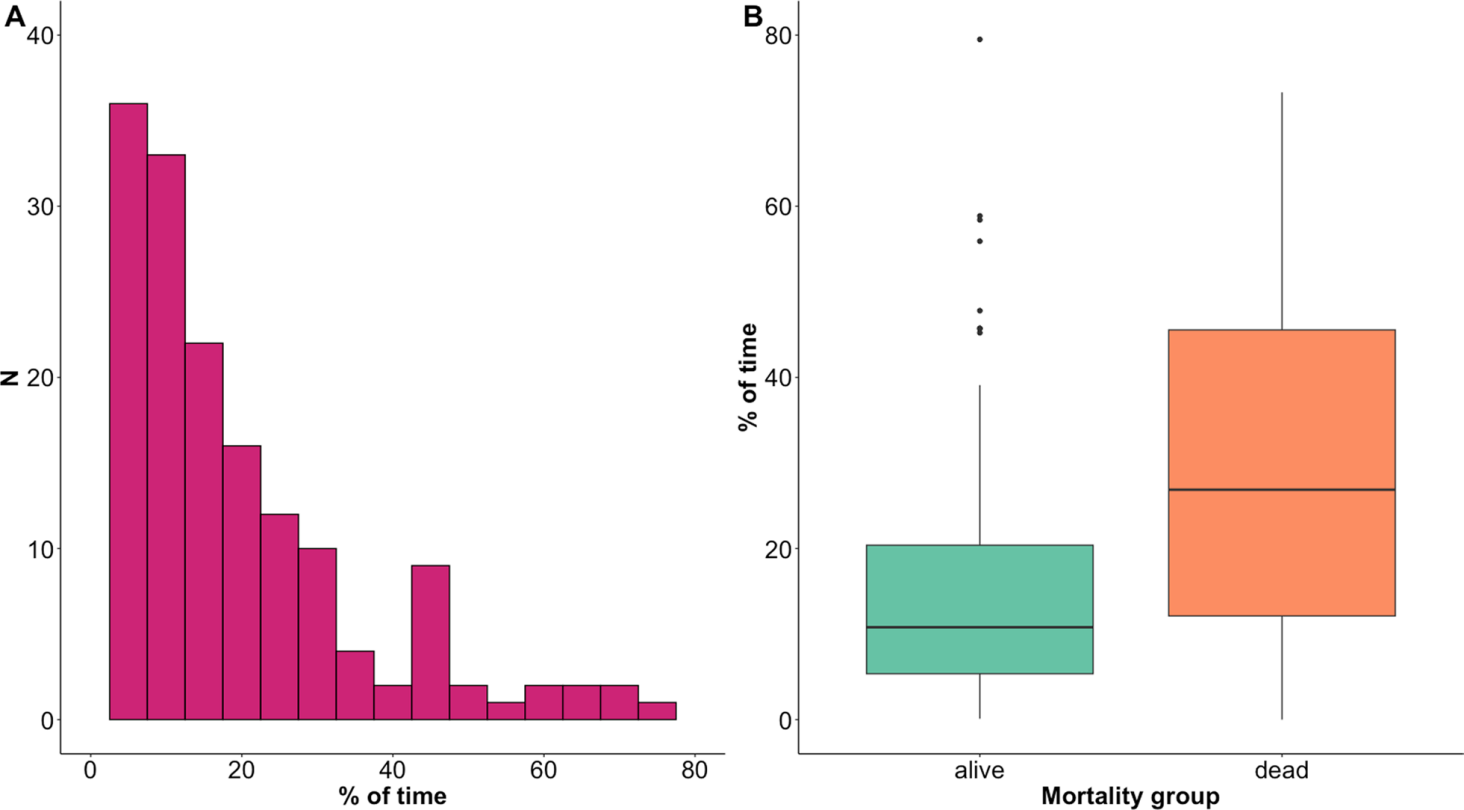
Percentage of time spent with CPP below LLR. Panel** A** shows the distribution of average values of percentage of time spent with CPP below LLR for the 7 days from the day of injury. Panel** B** shows difference in the metric when comparing mortality groups. The % of time spent with CPP < LLR is higher in patients who died (Mann-U test, *p* < 0.001) CPP: cerebral perfusion pressure; LLR: lower limit of reactivity; N: number of patients

**Fig. 4:**
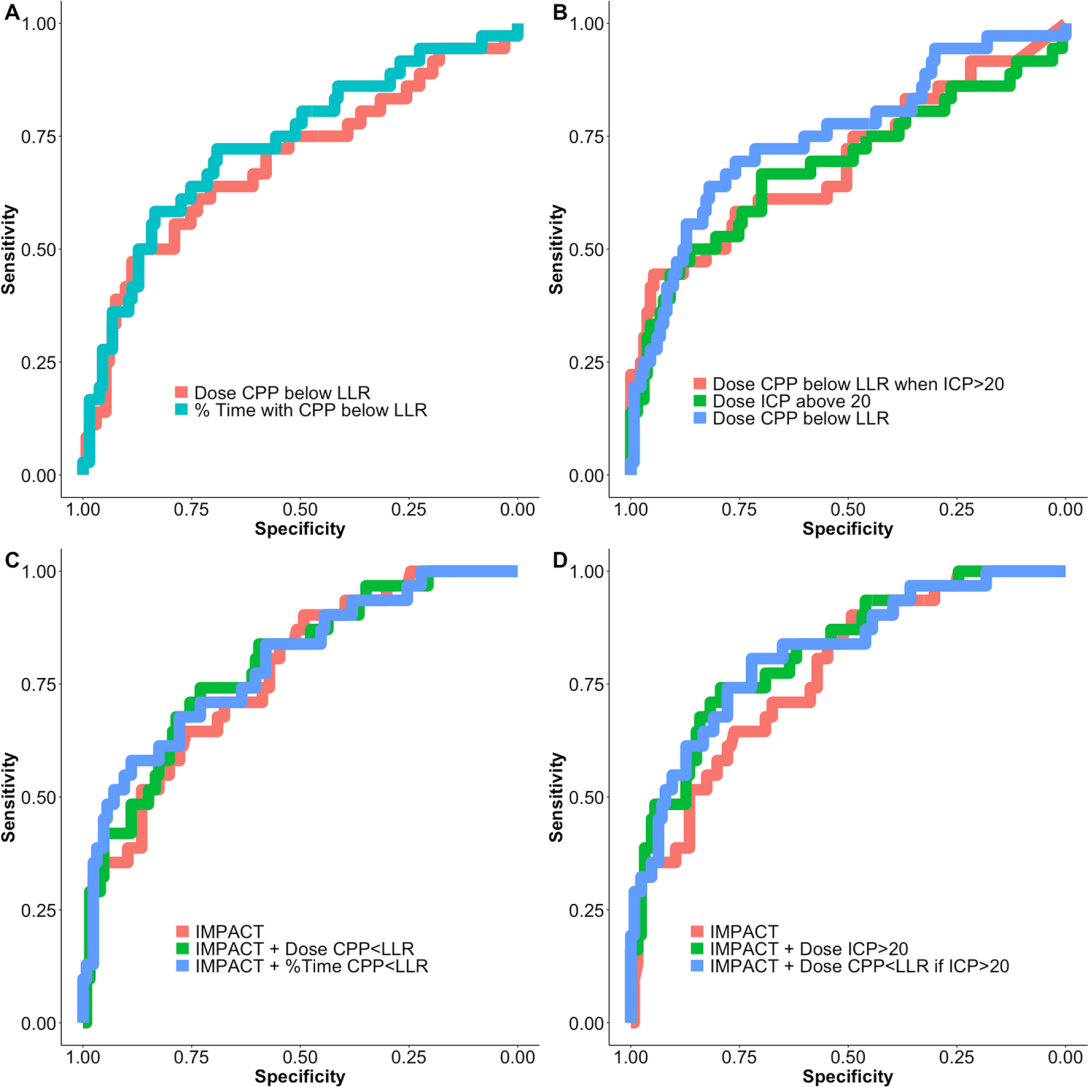
Univariate and multivariate logistic regression ROC curves for mortality prediction. A selection of Metrics that describe the relationship between LLR and CPP are considered in Panel** A** (univariate analysis) and** C** (multivariate analysis considering IMPACT core variables). Comparison with ICP is considered in Panel** B** (univariate analysis) and** D** (multivariate analysis considering IMPACT core variables). Models AUC 95% CI values are described in Tables 2 and 3. LLR: lower limit of reactivity; CPP: cerebral perfusion pressure; ICP: intracranial pressure; IMPACT: International Mission for Prognosis and Analysis of Clinical Trials. AUC: area under the curve

Median (IQR) LLR was higher (Mann-U test, *p* = 0.003) in patients who died (65 mmHg (58–72)) compared to patients who survived (59 mmHg (55–65)). Table 2 shows summary values for metrics that describe the relationship between CPP and LLR and metrics that describe the relationship between CPP, LLR and ICP. Results based on delta CPP below LLR are not reported, as the difference between outcome groups was clinically irrelevant, although statistically significant. For completeness, dose of ICP above different thresholds is also described. Mann-U *p* values and univariate logistic regression *p* values and AUC (95% CI) for mortality prediction show that all metrics can distinguish mortality groups. DeLong test did not suggest a difference between models. Figure 3 shows cohort distribution values of % of time spent with CPP below LLR and the relationship between mortality groups. Figure 4 shows receiver operating characteristics curves of univariate logistic models for LLR related variables both for the relationship with CPP (panel A) and for the relationship with ICP (panel B). The whole 7-day period from the day of injury was considered.

**Table 2:** Univariate analysis for mortality prediction

Variable	Median (*N* = 171)	IQR	Mann-U test* p* value	Univariate logistic regression *p* value	
					AUC (95% CI)
Relationship between CPP and LLR					
Dose of CPP below LLR (mmHg*h)	52.2	( 16.4–118.6)	< 0.001	0.002	0.69 (0.58–0.80)
Time with CPP below LLR (%)	12.8	( 6.4–25.2)	< 0.001	< 0.001	0.73 (0.63–0.83)
Relationship between CPP below LLR and high ICP					
Dose of CPP below LLR when ICP > 20 mmHg (mmHg*h)	5.0	( 0.8–18.9)	< 0.001	< 0.001	0.69 (0.58–0.80)
Dose of CPP below LLR when ICP > 22 mmHg (mmHg*h)	2.6	( 0.4–11.6)	< 0.001	< 0.001	0.69 (0.58–0.80)
Dose of CPP below LLR when ICP > 25 mmHg (mmHg*h)	1.1	( 0.0–8.2)	0.002	< 0.001	0.67 (0.56–0.78)
Dose of ICP above 20 mmHg (mmHg*h)	15.8	( 3.0–56.9)	< 0.001	0.003	0.69 (0.57–0.80)
Dose of ICP above 22 mmHg (mmHg*h)	9.5	( 1.5–31.9)	< 0.001	0.005	0.68 (0.56–0.79)
Dose of ICP above 25 mmHg (mmHg*h)	4.6	( 0.6–16.5)	0.002	0.009	0.67 (0.55–0.78)
Dose of ICP above 20 mmHg when CPP < LLR (mmHg*h)	3.2	( 0.3–13.8)	0.001	< 0.001	0.67 (0.56–0.79)
Dose of ICP above 22 mmHg when CPP < LLR (mmHg*h)	1.6	( 0.1–9.7)	0.002	< 0.001	0.67 (0.56–0.78)
Dose of ICP above 25 mmHg when CPP < LLR (mmHg*h)	0.6	( 0.0–5.4)	0.004	0.002	0.65 (0.54–0.77)

The table shows univariate analysis for mortality prediction for variables that describe the relationship between CPP and LLR and between CPP, LLR and ICP. Variables are considered for the whole 7-day period from the day of injury. DeLong test did not show superiority of any of the models considered. *p* values are not corrected for multiple comparisons

*CPP* cerebral perfusion pressure, *ICP* intracranial pressure, *LLR* lower limit of reactivity

Metrics that describe the relationship between CPP and LLR could distinguish outcome groups dichotomised for unfavourable vs favourable outcome. This analysis was not within the main objectives of this study, hence the results are reported in Additional file 1: Table 1S, Figs. 1S and 2S.

#### Multivariate analysis

When dose of ICP above 20 mmHg (*p* = 0.382) and dose of CPP below LLR when ICP is above 20 mmHg (*p* = 0.004) were considered in a multivariate logistic regression for mortality prediction (*p* < 0.001, AUC 0.7 (0.58–0.81)) without the IMPACT score ‘core’ covariates, only dose of CPP below LLR when ICP is above 20 mmHg had a significant impact.

AUC (95% CI), Akaike criterion (AIC) and *p* values for the models with IMPACT core variables as covariates considered in the analysis are reported in Table 3. Metrics that describe the relationship between CPP and LLR were significant when added to models with IMPACT core parameters, with and without ICP related variables. None of the considered models showed superiority at the DeLong test analysis. However, the model “IMPACT core + Dose CPP < LLR if ICP > 20” performed with the best combination of model evaluation metrics. AUC was 0.88 (0.81–0.94), the same as the model “IMPACT core + Dose ICP > 20”. AIC was the lowest (123.15) counting for almost 2 points difference form the model “IMPACT core + Dose ICP > 20 if CPP < LLR” which had an AIC of 125.12. Adjusted* R*^2^ was the highest (0.57), with 1% of improvement from model “IMPACT core + Dose ICP > 20” and “IMPACT core + Dose ICP > 20 if CPP < LLR”.

**Table 3:** Multivariate logistic regression analysis for mortality

Model	AUC (95% CI)	AIC	*p* value	Adjusted*R*^2^
IMPACT core	0.84 (0.78–0.91)	135.06	< 0.001	0.49
IMPACT core + Dose ICP > 20	0.88 (0.82–0.94)	125.12	< 0.001	0.56
IMPACT core + Dose CPP < LLR	0.86 (0.79–0.93)	128.69	< 0.001	0.54
IMPACT core + %Time CPP < LLR	0.85 (0.78–0.92)	130.46	< 0.001	0.53
IMPACT core + Dose CPP < LLR if ICP > 20	0.88 (0.81–0.94)	123.15	< 0.001	0.57
IMPACT core + Dose ICP > 20 if CPP < LLR	0.87 (0.81–0.94)	125.42	< 0.001	0.56

The table presents the multivariate logistic regression analysis for mortality prediction for variables that describe the relationship between CPP and LLR and between CPP, LLR and ICP, with IMPACT core covariates. The table shows only models where the tested variable added significant contribution in predicting mortality, when considered together with the IMPACT core variables. IMPACT: International Mission for Prognosis and Analysis of Clinical Trials

*ICP* intracranial pressure,* CPP* cerebral perfusion pressure,* LLR* lower limit of reactivity.* AIC*  Akaike Information Criterion, *AUC*  Area Under the Curve

Figure 4 shows receiver operating characteristics curves of multivariate logistic models for LLR related variables both for the relationship with CPP (panel C) and for the relationship with ICP (panel D) when added to IMPACT core variables. The whole 7-day period from the day of injury was considered.

### Outcome analysis stratified by the day post injury

**Fig. 5:**
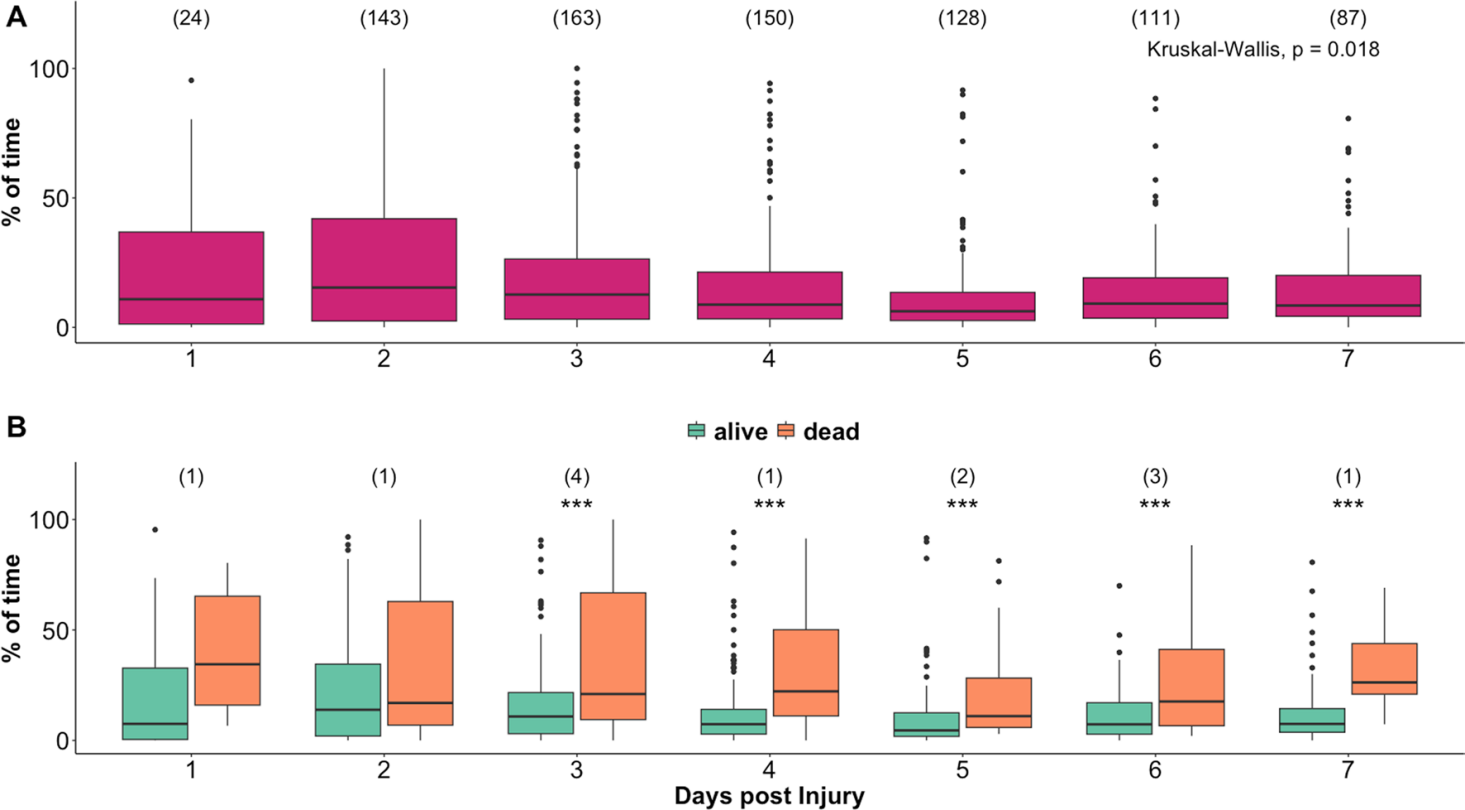
Relationship between daily percentage of time with CPP below LLR and outcome. Panel** A** shows boxplots of percentage of time with CPP below LLR for each day post injury. Numbers in brackets represent number of recordings available at each day. Panel** B** shows the relationship of the metric with mortality groups for each day. Black asterisks indicate statistically significant difference between mortality groups (Kruskal–Wallis test,* p* < 0.05). Numbers in brackets show the absolute number of patients that died at each day. A total number of 13 patients died within the first 7 days from the day of injury. CPP: cerebral perfusion pressure; LLR: lower limit of reactivity

Mortality prediction for multiple days post injury analysis showed similar results for all the metrics used to explore the relationship between CPP and LLR. Hence here we report only results for the relationship between daily % of time spent with CPP below LLR (Fig. 5). The relationship with dichotomised outcome becomes significant starting from the third day post injury (Kruskal–Wallis test, *p* < 0.05).

## Discussion

This is the first exploratory study that evaluates in details the lower limit of reactivity (LLR) in a multicentre cohort of traumatic brain injury (TBI) patients. LLR was assessed with an automated algorithm adapted for prospective use. We demonstrated that it is feasible to assess the lower limit of reactivity continuously in the majority of our cohort. We confirmed an earlier result [16] that the deviation of cerebral perfusion pressure (CPP) below LLR is associated with excess mortality.

The main finding is the validation of outcome predictive power of LLR in a multicentre cohort of TBI patients. Deviation of CPP below LLR was studied over the first days post injury, when the clinical management is time-critical. Dose of the amount of deviation of CPP below LLR and percentage of time with CPP below LLR were examined. Both metrics were significantly different between mortality groups as defined by GOSE at 6 months, also after adjusting for IMPACT covariates (Table 2, Fig. 4). This finding validates the results published by Donnelly et al. [16] who showed that in a single-centre cohort of TBI patients, the more time the patients spent with CPP below LLR, the higher the mortality. The next step in this direction will be to investigate the relationship between LLR time trend and admission and day-to-day ICU treatments and pathology progression. However, this type of analysis was beyond the scope of our work. Yet, our results highlight the importance of the relationship between LLR and outcome in TBI patients.

We observed that the relationship with survival becomes significant starting from the third day post injury (Fig. 5). This might be related to lower number of recordings available for day 1 (24 recordings). However, there was a similar number of recordings available on day 2 (142 recordings) and day 3 (172 recordings) suggesting that this effect is not likely to result purely from the statistical uncertainties. On the other hand, inflammation and vasogenic oedema are likely to become important mechanistic factors contributing a reduction in intracranial compliance and perhaps influencing CA over this timescale [34]. It is therefore plausible to assume that these mechanisms might be involved in the detrimental effect that ischaemic episodes of CPP lower than LLR have on the injured brain. Further research is needed for understanding the relationship between LLR and pathophysiological aspects of TBI, with a particular focus on the dynamic aspects of cerebral autoregulation and its determinants.

It is well acknowledged that intracranial hypertension is related to poor outcome in TBI patients [35]. The injured brain appears more vulnerable to ICP insults when autoregulation is impaired [24]. Moreover, cerebrovascular reactivity (as estimated by PRx) is independently associated with outcome over and above the IMPACT prognostic model covariates [26]. However, those studies looked at the impairment of vascular reactivity without distinguishing whether this happened below the lower limit or above the upper limit of reactivity. We also explored the relationship between CPP below LLR and high ICP and compared their ability in predicting outcome for mortality groups (Fig. 1, Table 2 and Fig. 4). The dose of ICP above different thresholds did not perform any better than the dose of CPP below LLR or the dose of CPP below LLR when ICP was above the threshold. Of note, the dose of ICP above threshold lost significance when considered together with the dose of CPP below LLR when ICP was above the threshold in a multivariate model (Results, Multivariate analysis section). This finding is in line with the current literature [24] and supports the concept that ICP insults are more detrimental when they cause cerebral ‘hypoperfusion’, as defined by episodes of CPP below LLR.

LLR could be calculated in 98% of patients of the CENTER-TBI cohort considered for this study. The yield of LLR was around 80% of CPP recorded time, similar to the yield performance of CPPopt in this cohort [23]. Both these findings are relevant for putative clinical application. They translate in possible availability of LLR automated assessment for majority of patients and for most of the time. The fact that these results were obtained with an automated algorithm that was adapted for bedside prospective use in TBI patients, strengthens the clinical relevance of our findings. A few considerations are worth mentioning for patients where LLR could not be calculated. These patients had very high values of ICP and completely lost cerebrovascular reactivity since the beginning of their recording (that is close to the beginning of their ICU admission) and had clearly un-survivable injuries. If autoregulation is completely lost, that is PRx is always at very high values, the relationship between PRx and CPP used for assessment of LLR should not be able to identify any levels of CPP corresponding to LLR. Even if from the mathematical point of view this could be feasible in certain cases, the physiological interpretation would be meaningless. Simply, at any value of CPP, autoregulation would be impaired[36]. The ability of disregarding possible mathematical outputs that would not have any physiological meaning, highlights the improvement in terms of reliability of this automated algorithm [22].

The range of LLR values over the first 7 days post injury covers a large span, as shown in Fig. 2. Current international guidelines[37] recommend a standard management of CPP between 60 and 70 mmHg (range highlighted in grey in Fig. 2). Hence, 60 mmHg could be considered a safe lower CPP limit, according to guideline recommendations. On the other hand, the distribution of the pressure reactivity based CPP lower limit shows that for only half of these patients LLR is lower than 60 mmHg. For the remaining half, average LLR is higher than 60, and even higher than 70 mmHg in 18% of cases. Keeping CPP within 60–70 mmHg might represent a risk for hypoperfusion episodes for those patients whose LLR is above this range. It is worth noting that average LLR values were higher in patients that died (likely from uncontrollable ICP). Even if this observation is based on a population level analysis and on a whole monitoring period, it supports the idea of a right-shift of the autoregulatory range or, possibly, a narrower autoregulatory range in this group of patients [21]. An individualised LLR-based CPP lower safety limit, continuously assessed at the bedside, and able to capture the dynamic aspect of autoregulation, could help in preventing brain hypoperfusion and possible ischaemic insults in TBI patients.

The role of LLR as a dynamic individualised CPP target in TBI patients remains uncertain. As opposed to the optimal cerebral perfusion pressure (CPPopt, the pressure in the middle of the autoregulatory plateau), the lower limit of autoregulation, or reactivity, is less investigated. From a practical point of view, targeting CPP at LLR as such might cause erroneous therapeutic management in TBI patients. Indeed, LLR represents an autoregulation based lower safety threshold for CPP, that means that CPP should be kept above at least LLR at all times. Instead, the advantage of having a continuous assessment of LLR at the bedside lies on the fact that the clinical team would be aware of the CPP range available for action. If both CPPopt and LLR were continuously available, and the range between CPPopt and LLR was wide, then aiming for CPP just above LLR (instead of the much higher CPPopt) could represent a safe and effective CPP management. This would spare vasopressors, fluid therapy, or sedation required to push arterial blood pressure higher or to decrease ICP. If, on the other hand, the autoregulatory range happened to be narrow, then aiming for the middle of the plateau, the CPPopt value, would be warranted. An additional (and most important) consideration is the uncertainty associated with LLR estimation and the fact that this quantity results from a superposition of vascular responses over a wider brain region, parts of which may well be under-perfused even when CPP is above LLR [41, 42]. Therefore CPP > LLR does not exclude the possibility of *focal* ischaemia. Hence, targeting CPPopt might still, for the moment, represent a safer option. However, LLR may represent an absolute region of danger. Further cautious investigation is required to highlight possible applications of LLR in TBI management.

### Limitations

We should admit that our analysis has some limitations. First, this study is a retrospective analysis. The cohort is limited compared to other TBI cohorts where autoregulation has been studied. EVD and DC patients were excluded, as special considerations are needed for the pressure reactivity index (PRx) calculations.

Our model assumes that threshold of PRx set for identifying loss of autoregulation, is the same for all patients. This might seem to breach the initial postulate to avoid ‘one size fit all’ policy and is an important methodological drawback of the LLR methodology, when compared to the CPPopt methodology. CPPopt does not depend on any subjective value or threshold, as it is identified by the optimum of the U-shape curve, which can correspond to any PRx value. The literature available does not provide with an ultimate gold standard threshold for defining LLR based on PRx in TBI patients [16, 38–41], and our goal was to validate our approach, rather than to suggest any new thresholds. Even though the threshold for PRx is fixed, it leads to different LLR values in different individuals. However, we advocate that methodological research is required to identify the LLR without the necessity of a fixed threshold.

In our current study we did not assess the physiological time variability of LLR and the patients’ LLR trajectories, along with the determinants involved in the changes of the time trends. Nor we included confounders like carbon dioxide and vasopressors in our multivariable analysis. These limitations warrant to be investigated in future studies.

Whether drops of cerebral perfusion pressure (CPP) below the continuously assessed PRx-based lower limit of autoregulation (LLR) truly translate into ischaemic insults in the human injured brain, remains to be established. Autoregulation is only one of the mechanisms involved in cerebral blood flow regulation. In fact, autoregulation works together with chemo-regulation, neuronal regulation and endothelium-dependent regulation. In this work, we focused on the association of autoregulation based CPP lower limit with outcome assessed at 6 months with the GOSE score. This information is certainly relevant in suggesting the possible impact of LLR in management of TBI patients. However, associations with cerebral ischaemic related variables that are close in time to the hypotensive episode, would provide proof of concept knowledge. Multimodality monitoring data derived from brain tissue oxygen and lactate/pyruvate ratio as well as brain damage biomarkers and imaging studies, might improve our understanding of the effect that episodes with CPP below LLR have. We did not perform such investigation. Future studies are required for addressing this issue.

## Conclusions

Using a multicentre prospective cohort, we confirmed that CPP below LLR during the first seven days post injury positively correlates with six-months mortality. This supports future investigations into personalised and dynamic CPP targets in TBI care.

### Supplementary Information

**Additional file 1.** Unfavourable outcome analysis.

## Data Availability

The data presented in this study can be accessed in the CENTER-TBI portal (https://www.center-tbi.eu/data).

## Abbreviations

TBI: Traumatic brain injury
CPP: Cerebral perfusion pressure
CA: Cerebral autoregulation
LLA: Lower limit of autoregulation
LLR: Lower limit of reactivity
ICP: Intracranial pressure
DC: Decompressive craniectomy
EVD: External Ventricular Drain
GCS: Glasgow Coma Scale
PRx: Pressure reactivity index
ABP: Arterial blood pressure
GOSE: Glasgow outcome score extended
IMPACT: International Mission for Prognosis and Analysis of Clinical Trials in TBI
ICU: Intensive Care Unit
