# Supplementary material for: The lower limit of reactivity as a potential individualised cerebral perfusion pressure target in traumatic brain injury: a CENTER-TBI high-resolution sub-study analysis
Source: Crit Care. 2023 May 20;27:194. doi: 10.1186/s13054-023-04485-8 (PMC10199598; doi:10.1186/s13054-023-04485-8)
Supplement: Supplementary file 1 — Additional file 1. Unfavourable outcome analysis. [file 13054_2023_4485_MOESM1_ESM.docx]

**Additional File 1**

**Unfavourable outcome analysis**

Table 1S shows summary values for metrics that describe the relationship between CPP and LLR and metrics that describe the relationship between CPP, LLR and ICP. Mann-U *p* values and univariate logistic regression *p*-values and AUC (95%CI) for unfavourable outcome prediction show that only metrics that describe the relationship between CPP and LLR, without considering the interaction with ICP, can distinguish outcome groups defined by unfavourable (GOSE <4) and favourable outcome.

Figure 1S panel A shows that the % of time spent with CPP below LLR is different between all outcome GOSE categories (Kruskall-Wallis, p<0.001). Figure 1S panel B shows boxplots of the relationship between unfavourable and favourable outcome groups.

Figure 2S shows that the relationship with dichotomised outcome becomes significant on the second day post injury.

**Table 1S. Univariate analysis for unfavourable outcome prediction.**

|  | | | | | Univariate logistic regression | |
| --- | --- | --- | --- | --- | --- | --- |
| Variable |  | Median | IQR | Mann-U test p-value | p-value | AUC (95% CI) |
| Relationship between CPP and LLR |  |  |  |  |  |  |
|  | Delta CPP below LLR (mmHg) | 0.8 | ( 0.3 - 1.7 ) | <0.001 | 0.003 | 0.68 (0.6-0.76) |
|  | Dose of CPP below LLR (mmHg*h) | 52.2 | ( 16.4 - 118.6 ) | <0.001 | 0.001 | 0.67 (0.59-0.75) |
|  | Time with CPP below LLR (%) | 12.8 | ( 6.4 - 25.2 ) | <0.001 | <0.001 | 0.68 (0.6-0.76) |
| Relationship between CPP below LLR and high ICP |  |  |  |  |  |  |
|  | Dose of CPP below LLR when ICP > 20 mmHg (mmHg*h) | 5.0 | ( 0.8 - 18.9 ) | 0.072 | 0.001 | 0.58 (0.49-0.67) |
|  | Dose of CPP below LLR when ICP > 22 mmHg (mmHg*h) | 2.6 | ( 0.4 - 11.6 ) | 0.097 | 0.002 | 0.57 (0.49-0.66) |
|  | Dose of CPP below LLR when ICP > 25 mmHg (mmHg*h) | 1.1 | ( 0.0 - 8.2 ) | 0.111 | 0.006 | 0.57 (0.48-0.66) |
|  | Dose of ICP above 20 mmHg (mmHg*h) | 15.8 | ( 3.0 - 56.9 ) | 0.067 | 0.022 | 0.58 (0.49-0.67) |
|  | Dose of ICP above 22 mmHg (mmHg*h) | 9.5 | ( 1.5 - 31.9 ) | 0.073 | 0.024 | 0.58 (0.49-0.67) |
|  | Dose of ICP above 25 mmHg (mmHg*h) | 4.6 | ( 0.6 - 16.5 ) | 0.091 | 0.024 | 0.58 (0.49-0.66) |
|  | Dose of ICP above 20 mmHg when CPP < LLR (mmHg*h) | 3.2 | ( 0.3 - 13.8 ) | 0.129 | 0.004 | 0.43 (0.34-0.52) |
|  | Dose of ICP above 22 mmHg when CPP < LLR (mmHg*h) | 1.6 | ( 0.1 - 9.7 ) | 0.098 | 0.007 | 0.57 (0.49-0.66) |
|  | Dose of ICP above 25 mmHg when CPP < LLR (mmHg*h) | 0.6 | ( 0.0 - 5.4 ) | 0.097 | 0.016 | 0.57 (0.49-0.66) |

The table shows the univariate analysis for unfavourable outcome prediction for variables that describe the relationship between CPP and LLR and between CPP, LLR and ICP. Variables are considered for the whole 7 days period from the day of injury. DeLong test did not show superiority of any of the models considered. CPP: cerebral perfusion pressure; ICP: intracranial pressure; LLR: lower limit of reactivity.

**Figure 1S. Percentage of time spent with CPP below LLR.** Panel A shows the distribution of average values of percentage of time spent with CPP below LLR for the 7 days from the day of injury over each GOSE category at 6 months. Panel B shows difference in the metric when at dichotomised outcome. The % of time spent with CPP<LLR is higher in patients that had unfavourable outcome (Mann-U test, p < 0.001) CPP: cerebral perfusion pressure; LLR: lower limit of reactivity.

**Figure 2S. Relationship between daily percentage of time with CPP below LLR and dichotomised outcome.** The figure shows boxplots of percentage of time with CPP below LLR and the relationship of the metric with outcome groups for each day. Black asterisks indicate statistically significant difference between mortality groups (Kruskal-Wallis test, p<0.05 (**) and p<0.001(***)). CPP: cerebral perfusion pressure; LLR: lower limit of reactivity.
